# The International Medical Annual and Practitioners’ Index

**Published:** 1900-08

**Authors:** 


The International Medical Annual and Practitioners' Index. A Work of Reference for Medical Practitioners. Eighteenth year. New York: E. B. Treat & Co., 241–243 West 23d Street. 1900.

The appearance of the Annual is looked forward to with much expectancy and pleasure, by all those acquainted with its variety of news and illustrations on subjects of great import to the medical mind. It serves to keep one abreast of the times in everything pertaining to the profession, and therefore is a welcome addition to the library.

The present volume is up to the high standard of preceding volumes and is full of new information and richly adorned with beautiful full-page plates.

Part I. is devoted to therapeutics and opens with a dictionary of new remedies and a review of therapeutic progress for 1899. The introduction of new organic compounds goes merrily on and such absurd extracts as neurine and choline, taken from the cerebrospinal fluid removed from cases of brain atrophy, and particularly from general paralysis of the insane; and the extract from the ciliary body of the ox are announced, the latter have been employed in cases of sympathetic ophthalmia. Among the newer drugs fully reviewed are heroin, holocaine, protargol and the various toxins. An interesting review of the progress in *x*-ray work during the past year then follows. The importance of *x*-ray work in the South African campaign is reviewed and its great importance in such cases fully noted.

Under Part II., new treatment is met, the familiar encyclopedic presentation of the progress achieved during the past year. Some of the subjects are treated at length and serve as monographs—such for instance is the article on Cancer by Keith W. Monsarrat, who goes quite extensively into the pathology of cancer and illustrates his findings by beautiful colored plates. For instance, the plate showing the method of spore formation in the organism is clear and nicely shown. The article on mycetoma is well described and accompanied by full-page illustrations.

The notes on legal decisions are of much interest to physicians, dentists and pharmacists and are prepared by W. A. Purrington of the Bellevue Hospital Medical College. Dr. Joseph Priestly contributes his annual review on the progress of sanitary science.

The closing chapter, as in the preceding numbers, gives a list of the principal medical works published during the year, chiefly American.

To all who have the preceding volume this number needs no introduction, to those not fortunate enough to possess them, the volume of 1900 is an excellent one to start with. W. C. K.

